# Light and Nutrient Dependent Responses in Secondary Metabolites of *Plantago lanceolata* Offspring Are Due to Phenotypic Plasticity in Experimental Grasslands

**DOI:** 10.1371/journal.pone.0136073

**Published:** 2015-09-03

**Authors:** Annegret Miehe-Steier, Christiane Roscher, Michael Reichelt, Jonathan Gershenzon, Sybille B. Unsicker

**Affiliations:** 1 Max Planck Institute for Chemical Ecology, Jena, Germany; 2 Max Planck Institute for Biogeochemistry, Jena, Germany; 3 UFZ, Helmholtz Centre for Environmental Research, Department of Community Ecology, Halle, Germany; Helmholtz Centre for Environmental Research (UFZ), GERMANY

## Abstract

A few studies in the past have shown that plant diversity in terms of species richness and functional composition can modify plant defense chemistry. However, it is not yet clear to what extent genetic differentiation of plant chemotypes or phenotypic plasticity in response to diversity-induced variation in growth conditions or a combination of both is responsible for this pattern. We collected seed families of ribwort plantain (*Plantago lanceolata*) from six-year old experimental grasslands of varying plant diversity (Jena Experiment). The offspring of these seed families was grown under standardized conditions with two levels of light and nutrients. The iridoid glycosides, catalpol and aucubin, and verbascoside, a caffeoyl phenylethanoid glycoside, were measured in roots and shoots. Although offspring of different seed families differed in the tissue concentrations of defensive metabolites, plant diversity in the mothers' environment did not explain the variation in the measured defensive metabolites of *P*. *lanceolata* offspring. However secondary metabolite levels in roots and shoots were strongly affected by light and nutrient availability. Highest concentrations of iridoid glycosides and verbascoside were found under high light conditions, and nutrient availability had positive effects on iridoid glycoside concentrations in plants grown under high light conditions. However, verbascoside concentrations decreased under high levels of nutrients irrespective of light. The data from our greenhouse study show that phenotypic plasticity in response to environmental variation rather than genetic differentiation in response to plant community diversity is responsible for variation in secondary metabolite concentrations of *P*. *lanceolata* in the six-year old communities of the grassland biodiversity experiment. Due to its large phenotypic plasticity *P*. *lanceolata* has the potential for a fast and efficient adjustment to varying environmental conditions in plant communities of different species richness and functional composition.

## Introduction

Plants growing in complex species rich communities not only have to compete with their neighbors for resources such as light and nutrients, but also have to fend off attackers such as herbivores and pathogens. To persist under these conditions, plants produce a large variety of chemical defense compounds. There is ample evidence that environmental parameters such as light, nutrients, ozone, CO_2_, water availability and temperature strongly affect the concentration of these defense compounds in plant tissues [1–4]. Nitrogen limitation for example can enhance the concentration of phenolic compounds [5,6] and other carbon based metabolites such as iridoid glycosides [7,8] and thus may have beneficial effects on plant defense against antagonists. Light availability also strongly affects the production of secondary metabolites such as terpenes and phenolics [9,10]. Apart from environmental factors, genetic variation can modify the phytochemistry of plants and their response to environmental change [11–13].

The environment experienced by individual plants in communities of increasing plant diversity in terms of species richness and functional composition varies in multiple abiotic and biotic factors such as plant neighbor identity, light and nutrient availability and interactions with pathogens and herbivores [14–17]. Therefore, it is most likely that plant species richness and community composition indirectly influence the defense chemistry of an individual plant species. However, experimental evidence for this assumption is scarce (but see [18,19]). A recent study in a grassland biodiversity experiment (Jena Experiment;[20]) revealed that plant diversity, namely species and functional group richness, in the surrounding plant community affects the investment of *Plantago lanceolata* L. (ribwort plantain) into direct defense metabolites, the iridoid glycosides catalpol and aucubin, six years after establishment of the experimental grasslands. Specifically, aucubin concentrations decreased with increasing species richness of the plant communities, where *P*. *lanceolata* was sampled, while catalpol concentrations increased with increased species richness and functional group number of the sampled plant communities [19]. *Plantago lanceolata* is known to exhibit phenotypic plasticity in growth-related morphological and chemical traits, but the results from studies addressing the role of phenotypic plasticity and genetic differentiation for adaptation to environmental variation in this species are inconsistent and depend on the studied traits [11,21,22]. Therefore, the observed variation in foliar iridoid glycoside concentrations in the biodiversity experiment may be the consequence of the different environmental conditions experienced by the plant individuals growing in communities of varying species richness and composition, but could also be due to genetic differentiation of *P*. *lanceolata* populations since their establishment from an identical seed source in the experimental communities.

The aim of the present study was to disentangle environmental and genetic effects on defense compounds in *P*. *lanceolata*. The most important defense metabolites in this species are the two iridoid glycosides, aucubin and catalpol, which are especially toxic to generalist herbivores and thus may help the plant reduce the loss of tissue important for carbon assimilation, nutrient uptake or reproduction [23–25]. Yet these compounds also function as stimulants for oviposition by specialist herbivores [26–28]. It is well known that specialized insects feeding on *P*. *lanceolata* can sequester iridoid glycosides for their own defense [29–31]. Less is known about the function of verbascoside, a caffeoyl phenylethanoid glycoside that is also highly abundant in *P*. *lanceolata*. A few studies suggest that verbascoside may play a role in defense against molluscs and that it has antibacterial and antifungal effects [32,33].

We sampled five seed families in each of ten experimental grasslands (Jena Experiment; [20]), and grew the offspring in a greenhouse experiment under controlled environmental conditions. Two levels of light and nutrient availability were applied because analyses of previous field data [19] have shown that these factors were most likely related to the differential investment of *P*. *lanceolata* into catalpol and aucubin in response to increasing plant diversity. After growing for thirteen weeks, plants were harvested, above- and belowground biomass was determined and the concentrations of aucubin, catalpol and verbascoside were measured to address the following questions: (1) Is variation in levels of iridoid glycosides in response to increasing plant diversity attributable to genetic differentiation due to diversity-induced different selection or to variation in the actual growth environment? (2) What are the single and combined effects of light and nutrient availability on iridoid glycosides and verbascoside concentrations in *Plantago lanceolata*? (3) Are changes in levels of defense metabolites associated with plant biomass?

## Materials and Methods

### Study organism

*Plantago lanceolata* L. (ribwort plantain; Plantaginaceae) is a short-lived perennial rosette plant species with a worldwide distribution that typically belongs to European meadow communities. It is wind-pollinated or occasionally insect-pollinated, incompletely self-incompatible and may also reproduce vegetatively by forming new rosettes from axillary buds [34–36].

### Field site: the Jena Experiment

Seed material was collected from plants growing in a large-scale biodiversity experiment (Jena Experiment; [20]), which is located in the floodplain of the river Saale near Jena, Germany (50°55`N, 11°35`E, 130 m a.s.l.). The field site was rented by the research consortium of the Jena Experiment from an agricultural collective. No specific permission was required for the described study. Our study did not involve endangered species and the field site is not under nature protection. The area around Jena has a mean annual air temperature of 9.3°C; mean annual precipitation amounts to 587 mm [37]. The Jena Experiment is based on a pool of 60 plant species common to Central European mesophilic grasslands (Arrhenatherion community, [38]), which were divided into four functional groups: 16 grasses, 12 legumes, 12 small herbs and 20 tall herbs [20]. In total, the Jena Experiment comprises 82 plots of 20 x 20 m size that cover a gradient in species richness (1, 2, 4, 8, 16, and 60) and functional group number (1 to 4) in a near-orthogonal design. Species composition for each species richness x functional group number combination was chosen by random draws with replacement from the respective functional groups ensuring that replicates per species-richness level did not represent identical species compositions (with exception of the 60-species mixture). Plots were arranged into four blocks parallel to the river to account for a gradient in soil texture. The biodiversity experiment was established by sowing in spring 2002. Seed material was purchased from a commercial supplier (Rieger-Hofmann GmbH, Blaufelden Raboldshausen, Germany). The originally sown species combinations have been maintained by weeding twice per year (early April, July). All plots were mown each year in June and September and were not fertilized.

### Plant material

In July 2008, seeds from fruiting plants (= mothers) of *P*. *lanceolata* were collected in 10 experimental grassland plots: one *P*. *lanceolata* monoculture, 2-, 4-, 8-, and 16 species-mixtures each with two replicates (representing a species combination without and a species combination with legumes respectively), and one 60-species mixture (see S1 File Table A for mixture composition). In each plot, seeds of five randomly chosen mother individuals (= seed families) were collected at a minimum distance of 0.5 m between different mother individuals. Seeds were stored at –20°C to maintain their viability until the start of the experiment.

### Cultivation and experimental treatments

Seeds were washed with 70% ethanol and flushed with distilled water for sterilization. Washed seeds were sown in pots filled with commercially available substrate (Tonsubstrat, Klasmann-Deilmann GmbH, Geeste, Germany) for germination on the 1^st^ October 2009. Two weeks after germination, 12 seedlings of each seed family were transplanted into 0.5 L plastic pots (10 cm diameter) filled with a mixture of sand and nutrient poor soil (9:1; Fruhstorfer Nullerde, Hawita Gruppe GmbH, Vechta, Germany). Plants were grown at 21°C with 14 h of light per day. Five days after transplantation all plants were fertilized with 5 ml of a 200% Hoaglands solution [39]. Twenty-two days after transplanting, light and nutrient treatments, each with two levels, in a factorial design were initiated. Each seed family was represented by three offspring per treatment. In total, the experiment comprised 600 plants (three offspring individuals per mother, five mother plants from each of ten grassland plots and four different treatments). Light availability was reduced for half of the plants by shading them with a green semi-transparent plastic net (polyethylene, aperture size 2 x 10 mm, Hermann Meyer KG, Rellingen, Germany) clamped to a metal frame at 150 cm height. Light intensity in the high light treatment was 175 μmol s^-1^ m^-2^ simulating moderate shade compared to photosynthetically active radiation above the canopy (693 ± 214 μmol s^-1^ m^-2^ based on day-time means from early May to late August 2007 measured in the field [40], and in the low light treatment it was 35 μmol s^-1^ m^-2^, reflecting deep shade conditions. The manipulation of light availability was crossed with two levels of nutrient availability. Plants growing under high-nutrient conditions were fertilized twice a week with 11.5 mL of a 200% Hoaglands solution, while low-nutrient plants were fertilized with the same volume of a 50% Hoaglands solution ([35], see S1 File Table B).

To control for effects of shading on air temperature and humidity, both were monitored in the shaded and non-shaded treatment for one week using data loggers (EL-USB-2, Farnell GmbH, Oberhaching, Germany). Differences in air temperature and humidity were minor between the shade treatments: 0.8 K difference in air temperature (= 21.3°C vs. 20.5°C) and 2.3% difference in humidity (= 55.1% vs. 52.8% in the non-shaded and shaded treatment respectively). Plants were moved weekly within each treatment block. Due to a slight mildew infestation all plants were treated twice with a fungicide on 17^th^ November and 1^st^ December 2009 (Baymat, COMPO GmbH, Münster, Germany).

### Measurement of leaf gas exchange

One week before the plants were harvested (4^th^ to 9^th^ January 2010), leaf gas exchange was measured on a fully expanded leaf of the cultivated offspring of each of three seed families originating from the monoculture and the 60-species mixture, and therefore representing the extremes of the species-richness gradient of the biodiversity experiment, in each treatment (= 72 plants in total) to evaluate the effects of light and nutrient availability on carbon assimilation of *P*. *lanceolata*. Light response curves were measured with a LICOR-6400 Portable Photosynthesis System equipped with a 6400-02B LED light source (LI-COR, Lincoln, USA). The reference CO_2_ concentration was held constant at 380 μmol mol^-1^ and measurements were taken at 1000, 1200, 1500, 1800, 1500, 1200, 1000, 900, 800, 600, 400, 300, 100, 50, 25 and 0 μmol m^-2^ s^-1^ when the sample leaf was equilibrated for at least 60 s under each light step. Light intensity was increased at the beginning of each light response curve to avoid photoinhibition of shaded plants. Leaf temperature was held constant at 20°C. Light-saturated photosynthetic rate (A_max_; μmol CO_2_ m^-2^ s^-1^) was calculated using the hyperbolic tangent function by Jassby and Platt [36].

### Plant sampling

Thirteen weeks after the initiation of the light and nutrient treatments, all plants were harvested from 11^th^ to 15^th^ January 2010. Plants were cut at 1 cm above ground level. Roots were washed in a sieve (0.5 mm mesh size) to remove the substrate. All samples were flash-frozen in liquid nitrogen and stored at -80°C until freeze-drying. Lyophilized plant material was weighed and ground to a fine powder using a ball mill (Skandex SO-10 m, Fluid management B.V., Sassenheim, Netherlands) prior to further processing for chemical analyses.

### Chemical analyses

The offspring of each of three seed families originating from the monoculture and the 60 plant species mixture plot (= 72 samples in total) were analyzed for nitrogen and carbon concentrations. Approximately 20 mg of homogenized leaf and root material respectively were analyzed with an elemental analyzer (Vario EL, Elementar Analysensysteme GmbH, Hanau, Germany). Root samples of plants originating from seed families collected in monoculture and the 60-species mixture and leaf samples of all plants were analyzed for iridoid glycosides and verbascoside concentrations; 20 mg of leaf and root material were extracted in 1 ml 70% methanol on a shaker for 30 min at room temperature and centrifuged 10 min (3200 rpm). Afterwards, 200 μl of the supernatant were diluted with 600 μl deionized water and analyzed directly with high performance liquid chromatography using an Agilent 1100 Series HPLC System with an autosampler and diode array detector (Agilent Technologies, Waldbronn, Germany) employing a Nucleodur Sphinx RP-column (250 x 4.6 mm, 5 μm, Macherey-Nagel, Düren, Germany) and a 0.05% trifluoroacetic acid (solvent A)—acetonitrile (solvent B) gradient (flow rate 1ml min^-1^, injection volume 20 μl at 25°C, elution gradient: 0–10% B (10 min), 10–40% B (10 min), 40–100% B (0.1 min), 100% B (1.9 min), 100–10% B (0.1 min) and 10% B (4.9 min). Aucubin, catalpol and verbascoside were quantified by comparing the peak areas at 200 nm for catalpol and aucubin and at 270 nm for verbascoside against an external standard curve (catalpol standard: Wako Pure Chemical Industries, Ltd.; Osaka, Japan; aucubin standard: Carl Roth GmbH, Karlsruhe, Germany; verbascoside standard: Extrasynthèse, Genay, France).

### Statistical analysis

Linear mixed-effects models using the package *lme4* [41] of the statistical software R3.1.1. [42] were applied to analyse the effects the maternal plant identity (= seed family), the experimental factors of the origin environment in the biodiversity experiment (plant species richness, legume presence), the experimental treatments in the greenhouse experiment (light and nutrient availability) on variation in the measured variables. We considered seed family nested in origin plot as random effects, and species number (log-linear term), presence-absence of legumes, light and nutrient levels and the interaction between the latter as fixed effects. To control for possible genotype x environment interactions, we also included the interaction between seed family and growth environment (i.e. light and nutrient availability) in our models. Models for concentrations of secondary metabolites in the roots, photosynthesis and N concentrations did not include the experimental factors of the biodiversity experiment (species richness, legume presence-absence) because these measurements only included seed families originating from two plots (monoculture, 60-species mixture). Model selection was based on Akaike’s information criterion (AIC; [43] Following the recommendation of Zuur et al. [44] we started with a model with the maximal set of fixed effects and first decided about the adequate structure of the random effects which should be included in the final model. We fitted alternative models with (i) origin plot, (ii) seed family nested in origin plot, and interactions between the random seed family effect and (iii) light, (iv) nutrients, (v) light and nutrients, or (vi) light x nutrients. In a second step we selected to most parsimonious set of fixed effects. Finally, the *ghlt* function in the R package *multcomp* [45] was used for Tukey`s HSD test to identify differences between the different combinations of the light x nutrient treatments using the respective structure of random effects for each response variable. All variables except for A_max_, leaf nitrogen concentrations and root verbascoside concentrations were log-transformed to meet the assumptions of statistical modelling.

## Results

### Iridoid glycosides and verbascoside in *Plantago lanceolata*

**Fig 1:**
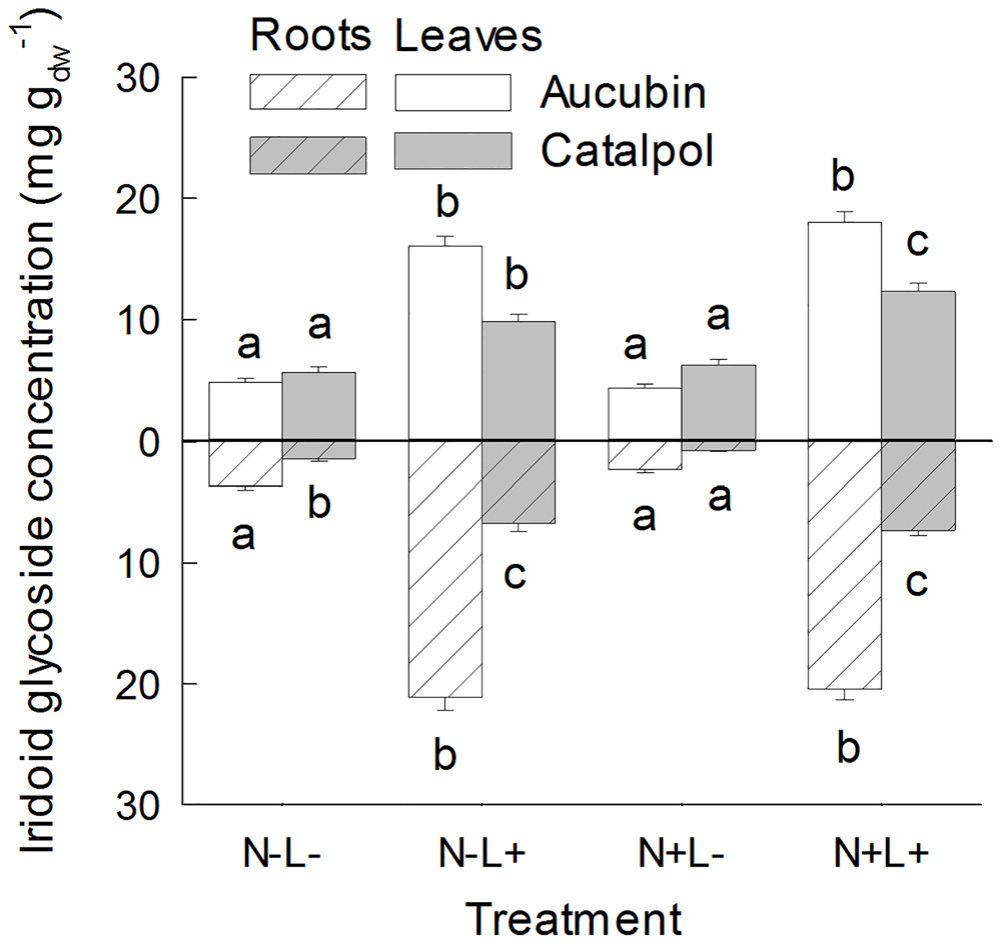
Aucubin and catalpol concentrations in leaves and roots of *Plantago lanceolata*. Plants were grown at different light and nutrient availability (N-: low nutrient, N+: high nutrient, L-: low light, L+: high light). Values are means across all plants per treatment (+ 1 SE). Results of Tukey`s test applied to test for significant differences between different light x nutrient treatments are indicated with letters.

On average the concentration of foliar iridoid glycosides was 19.2 mg g_dw_^-1^ (± SE 0.66). The iridoid glycoside concentration in the roots was 15.8 mg g_dw_^-1^ (± SE 1.19). The concentrations of total iridoid glycosides, aucubin and catalpol in leaves varied between seed families. Catalpol concentrations in the roots also depended on seed family identity, while we did not detect an effect of seed family identity on total iridoid glycoside and aucubin concentrations in the roots (Table 1). Total iridoid glycosides, aucubin and catalpol concentrations in leaves did not depend on species number and presence of legumes in the plots of origin (Table 1). High light conditions had positive effects on the concentrations of aucubin and catalpol in both above- and belowground plant tissue (Fig 1, Table 1).

**Table 1:** Summary of mixed-effects model analyses of iridoid glycoside and verbascoside concentrations in leaves and roots of *Plantago lanceolata*. Plants were grown at two different levels of nutrient and light availability and originated from seed families collected in experimental communities of different plant diversity. Root samples were analysed for offspring originating from three seed families taken from the monoculture and a 60-species mixture.

Source of variation	Total iridoid glycosides	Aucubin	Catalpol	Verbascosides
**Leaves**				
AIC	1284.1	1182.2	1279.4	1141.4
**Fixed effects**				
Intercept	2.123	1.491	1.557	2.255
*Origin environment*			--	--
Species richness	--	--	--	--
Legume	--	--	--	--
*Growth environment*				
Nutrients	--	*-0*.*100*	0.154	-0.394
Light	1.068	1.169	0.629	1.519
Nutrients x Light	--	0.225	--	--
**Random effects**				
Seed family (SF)	0.211	0.177	0.272	0.143
SF x Nutrients	--	--	--	--
SF x Light	--	--	--	--
SF x Nutrients x Light	--	--	--	--
Residual	0.698	0.640	0.685	0.622
**Roots**				
AIC	732.5	33.3	184.8	997.4
**Fixed effects**				
Intercept	3.997	2.046	0.524	48.086
*Growth environment*				
Nutrients	--	--	*-0*.*445*	--
Light	23.735	1.166	1.264	--
Nutrients x Light	--	--	0.661	--
**Random effects**				
Seed family (SF)	<0.001	<0.001	0.290	8.330
SF x Nutrients	--	--	0.254	--
SF x Light	--	--	--	8.912
SF x Nutrients x Light	--	--	--	--
Residual	5.078	0.269	0.468	14.647

Significance of seed family (SF) and interactions of seed family with the experimental factors (SF × Nutrients, SF × Light, SF × Nutrients × Light) were assessed based on the full fixed effect model. Afterwards, the set of fixed effects containing all significant predictors was determined by stepwise inclusion and model comparison. Estimated coefficients and AIC are given for the resulting best model. Intercept and slopes respectively are shown for the fixed effects and estimated standard deviations are given for the random effects.

While leaf aucubin concentrations were nearly four times higher under high light than under low light conditions, leaf catalpol concentrations had twice the concentrations under high light compared to low light conditions. Root aucubin and catalpol concentrations were seven times higher under high light than under low light conditions.

Nutrient addition also had positive effects on catalpol concentrations in leaves. The effects of nutrient addition on catalpol concentrations in the roots depended on the identity of the seed family (significant interaction SF x nutrients, Table 1) and varied with light availability. When plants were grown under low light conditions, catalpol concentrations in the roots were reduced by half in the high nutrient treatment, while nutrient availability had minor effects under high light conditions.

Effects of nutrient addition on foliar aucubin concentration depended on light availability (significant interaction nutrient x light). Aucubin concentrations were on average 12% higher in the high nutrient treatment than in the low nutrient treatment when plants were grown under high light conditions, while aucubin concentrations were on average 8% lower in the high nutrient treatment than in the low nutrient treatment when plants were grown under low light conditions although the group means between low and high light conditions did not differ significantly (Fig 1). Aucubin concentrations in the roots were not affected by nutrient availability.

**Fig 2:**
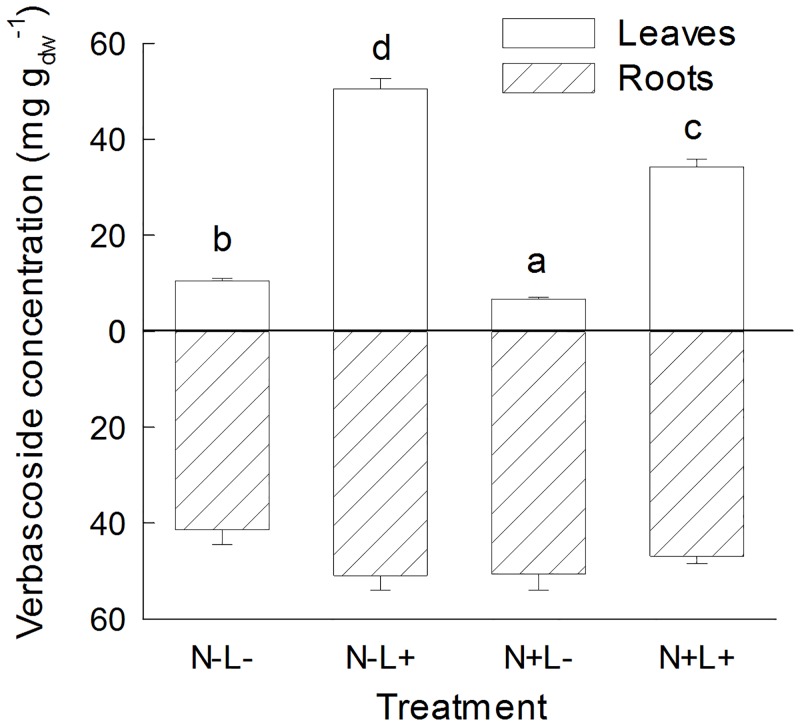
Verbascoside concentrations in leaves and roots of *Plantago lanceolata*. Plants were grown at different light and nutrient availability (N-: low nutrient, N+: high nutrient, L-: low light, L+: high light). Values are means across all plants per treatment (+ 1 SE). Results of Tukey`s test applied to test for significant differences between different light x nutrient treatments are indicated with letters.

Offspring of different seed families differed in the concentrations of verbascoside in above- and belowground plant tissue and the effects of light availability on verbascoside concentrations in the roots depended on seed family identity. The presence of legumes or number of species in the plots of origin did not affect verbascoside concentrations in leaf and root tissue of the offspring (Table 1). The foliar concentration of verbascoside was increased under high light conditions and decreased in the high nutrient treatment (Table 1) resulting in the highest leaf verbascoside concentrations under high light/low nutrient conditions and the lowest leaf verbascoside concentrations under low light/high nutrient conditions (Fig 2) The concentrations of verbascoside in the roots were not affected by the experimental conditions (Table 1, Fig 2).

### Biomass, rates of photosynthesis and tissue nitrogen concentrations

**Fig 3:**
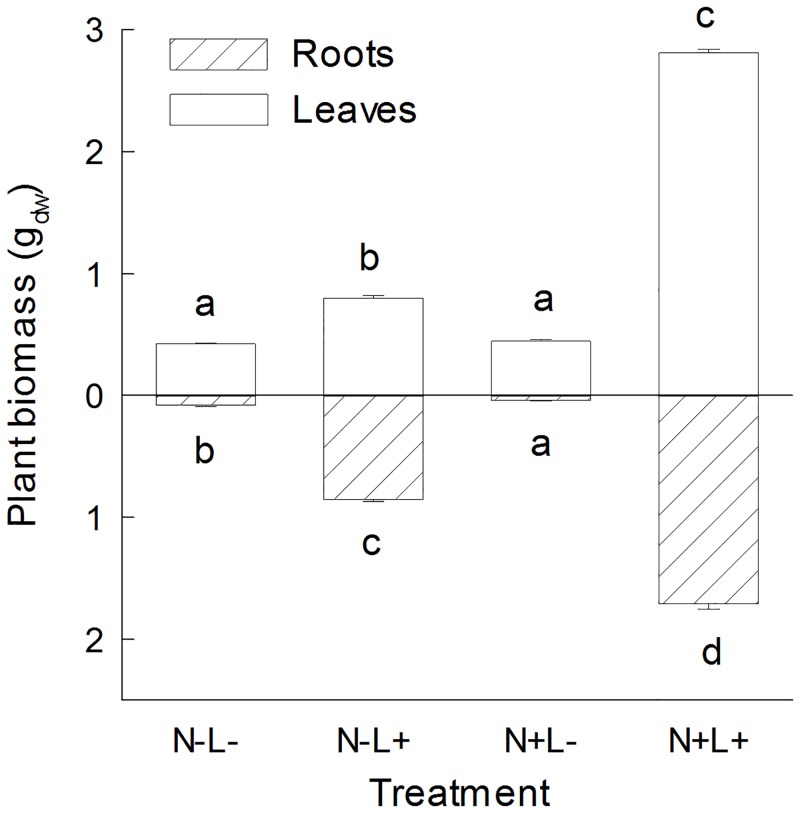
Above- and belowground biomass of leaves and roots of *Plantago lanceolata*. Plants were grown at different light and nutrient availability (N-: low nutrient, N+: high nutrient, L-: low light, L+: high light). Values are means across all plants per treatment (+ 1 SE). Results of Tukey`s test applied to test for significant differences between different light x nutrient treatments are indicated with letters.

Leaf and root biomass of *P*. *lanceolata* plants was significantly affected by light and nutrient availability and the interaction between both factors (Table 2). Light had a positive impact on plant biomass. Nutrient availability had large positive effects on leaf and root biomass under high light availability, while nutrient addition did not increase leaf biomass under low light conditions and fertilized plants even produced less root biomass than unfertilized plants under low light availability (Fig 3, Table 2). The impact of light availability (and nutrient availability in case of leaf biomass) also varied dependent on seed family identity.

**Table 2:** Summary of mixed-effects model analyses of leaf and root biomass and the shoot: root ratio measured for *Plantago lanceolata*. Plants were grown at two different levels of nutrient and light availability and originated from seed families collected in experimental communities of different plant diversity.

Source of variation	Leaf biomass	Root biomass	Shoot:root ratio
AIC	-128.8	440.9	255.7
**Fixed effects**			
Intercept	6.053	4.112	1.936
*Origin environment*			
Species richness	--	--	--
Legume	-0.012	--	--
*Growth environment*			
Nutrients	0.012	-0.428	0.439
Light	0.623	2.617	-1.993
Nutrients x Light	1.249	1.104	0.147
**Random effects**			
Seed family (SF)	<0.001	0.158	0.121
SF x Nutrients	<0.001	--	--
SF x Light	0.196	0.141	0.125
SF x Nutrients x Light	0.189	--	--
Residual	0.199	0.330	0.278

Significance of seed family (SF) and interactions of seed family with the experimental factors (SF × Nutrients, SF × Light, SF × Nutrients × Light) were assessed based on the full fixed effect model. Afterwards, the set of fixed effects containing all significant predictors was determined by stepwise inclusion and model comparison. Estimated coefficients and AIC are given for the resulting best model. Intercept and slopes respectively are shown for the fixed effects and estimated standard deviations are given for the random effects.

Plants cultivated under high light and high nutrient conditions had approximately 8.5 fold higher total biomass (4.5 g_dw_, SE ± 0.06) than plants grown in the low light treatment under high or low nutrient conditions (0.5 g_dw_, SE ± 0.02 at low nutrient availability; 0.5 g_dw_, SE ± 0.01 at high nutrient availability) and 2.6 fold higher than plants grown under high light and low nutrient conditions (1.7 g_dw_, SE ± 0.03). The ratio of leaf to root biomass also differed in response to light and nutrient availability. Plants grown under high light conditions had a greater fraction of root biomass. This was also true for plants grown under low nutrient conditions (Table 2). The significant interaction between both experimental factors implies that the combination of low light supply and high nutrient availability had the most crucial effect on a high ratio of leaf to root biomass.

**Fig 4:**
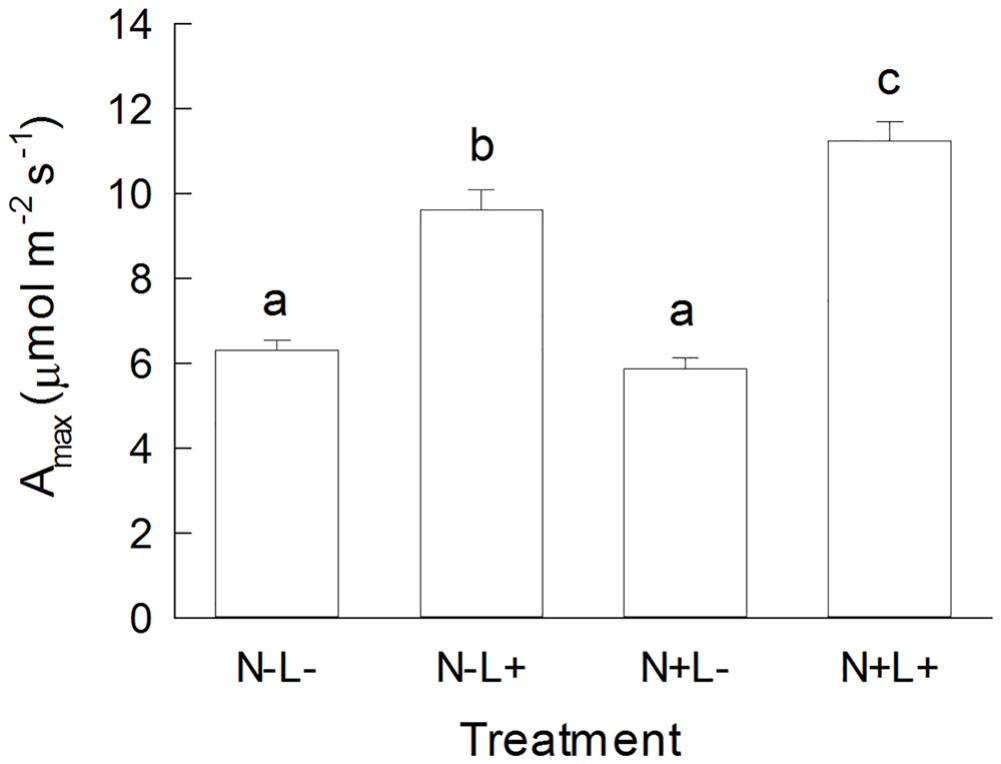
Maximum rate of photosynthesis (A_max_) of *Plantago lanceolata*. Plants were grown at different light and nutrient availability (N-: low nutrient, N+: high nutrient, L-: low light, L+: high light). A_max_ is measured as CO_2_ uptake (μmol m^-2^ s^-1^). Values are means across all plants per treatment (+ 1 SE). Results of Tukey`s test applied to test for significant differences between different light x nutrient treatments are indicated with letters.

**Fig 5:**
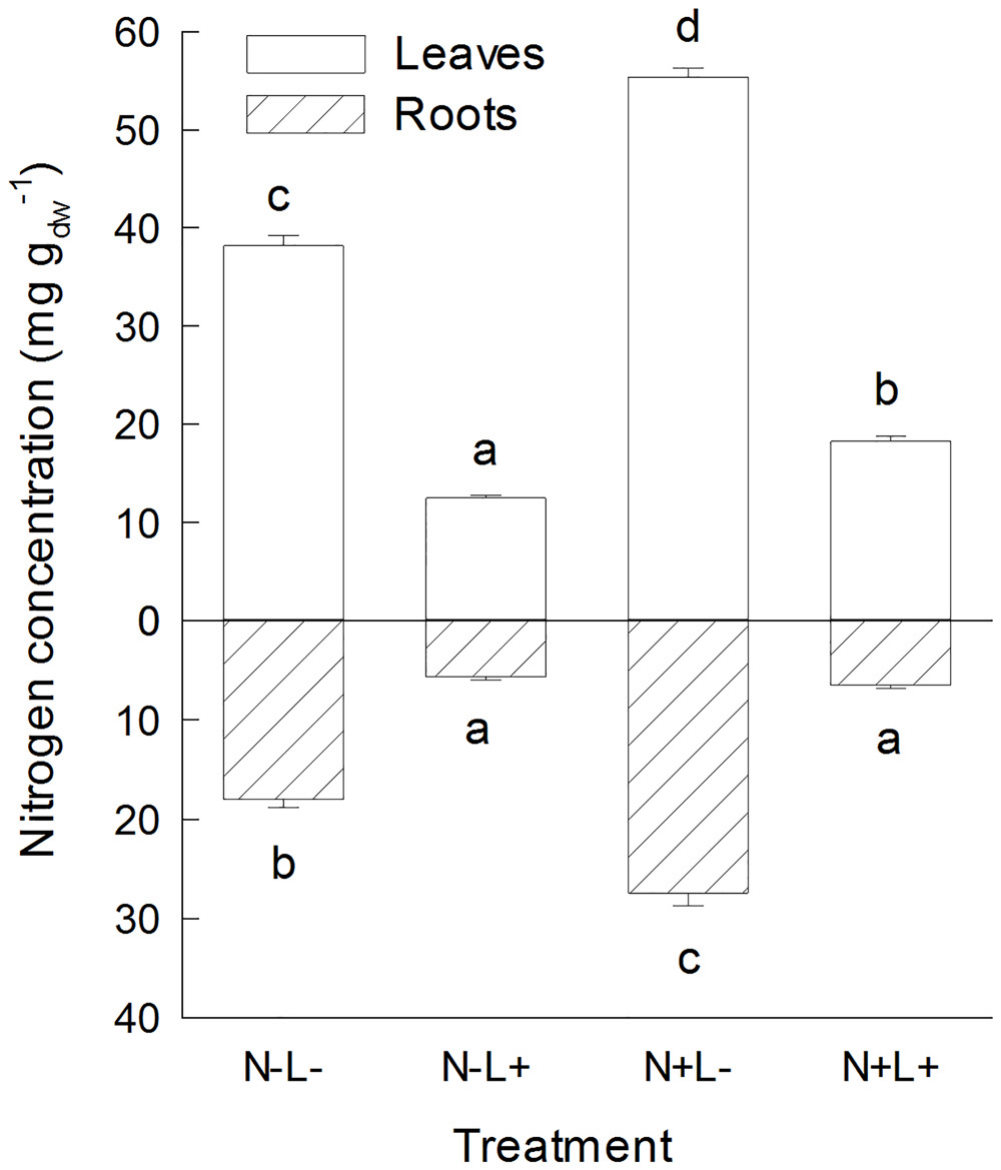
Nitrogen concentrations in leaves and roots of *Plantago lanceolata*. Plants were grown at different light and nutrient availability (N-: low nutrient, N+: high nutrient, L-: low light, L+: high light). Values are means across all plants per treatment (+ 1 SE). Results of Tukey`s test applied to test for significant differences between different light x nutrient treatments are indicated with letters.

On average, the light-saturated rate of photosynthesis (A_max_) in the offspring of *P*. *lanceolata* derived from the monoculture and a 60-species mixture was higher in plants grown in the high light treatment. Nutrient availability affected A_max_ positively under high light conditions (Fig 4, Table 3). The impact of light availability on A_max_ varied dependent on seed family identity (Table 3). Nitrogen concentrations in leaves and roots were positively influenced by nutrient supply but decreased under high light conditions (Fig 5, Table 3). While the effects of light and nutrient availability on leaf nitrogen concentrations differed between seed families, root nitrogen concentrations did not vary among seed families.

**Table 3:** Summary of mixed-effects model analyses of maximum rates of photosynthesis (A_max_) and nitrogen concentrations in leaves and roots of *Plantago lanceolata*. Plants were grown at two different levels of nutrient and light availability and originated from three seed families taken from the monoculture and a 60-species mixture.

Source of variation	A_max_	Leaf nitrogen	Root nitrogen
AIC	273.3	371.2	-7.6
**Fixed effects**			
Intercept	6.309	38.177	2.872
*Growth environment*			
Nutrients	-0.430	17.176	0.415
Light	3.303	-25.711	-1.161
Nutrients x Light	2.056	-11.370	-0.271
**Random effects**			
Seed family (SF)	0.018	3.402	0.037
SF x Nutrients	--	2.677	--
SF x Light	0.932	4.301	--
SF x Nutrients x Light	--	4.496	--
Residual	1.365	2.215	0.208

Significance of seed family (SF) and interactions of seed family with the experimental factors (SF × Nutrients, SF × Light, SF × Nutrients × Light) were assessed based on the full fixed effect model. Afterwards, the set of fixed effects containing all significant predictors was determined by stepwise inclusion and model comparison. Estimated coefficients and AIC are given for the resulting best model. Intercept and slopes respectively are shown for the fixed effects and estimated standard deviations are given for the random effects.

## Discussion

In this study we examined whether (1) varying levels of defense compounds in plants growing in plant communities of different plant diversity are due to genetic differentiation or phenotypic plasticity, and (2) how the availability of light and nutrients affect the investment into chemical defense compounds in *Plantago lanceolata*. Our study was motivated by the observation that changes in plant species richness and the presence-absence of legumes in the experimental plant communities of the “Jena Experiment” [20] influenced concentrations of iridoid glycosides in leaves of *P*. *lanceolata* [19]. Our greenhouse study revealed that the offspring from seed families of *P*. *lanceolata* plants growing in plant communities differing in plant diversity for more than six years contained different levels of iridoid glycosides and verbascoside. However, these differences were independent of plant diversity in terms of plant species richness and functional group composition at the original grassland plots in the biodiversity experiment. In contrast, the experimentally applied environmental conditions (two levels of light intensity and nutrient availability designed to simulate conditions in the different plant communities using data from measured natural light intensities and leaf nitrogen concentrations in the biodiversity experiment [19,40]) had strong effects on the levels of defense metabolites in roots and shoots of *P*. *lanceolata*. The variation in iridoid glycosides and verbascoside in *P*. *lanceolata* observed in communities of varying plant diversity and concomitant differences in environmental regimes is therefore attributable to phenotypic plasticity rather than the selection of genotypes specifically adapted to the growth conditions in plant communities of increasing diversity. A recent study by Zuppinger-Dingley and colleagues [46] revealed that selection for niche differentiation in response to plant species diversity in the experimental grassland communities of the Jena Experiment increases biodiversity effects 8 years after the establishment of this long-term experiment. In this study differences in the metabolic profiles of plant individuals originating from monocultures *versus* mixtures of four and more plant species were observed. Selection for plant resistance traits however were not investigated in this study [46].

### *Plantago lanceolata* exhibits phenotypic plasticity in morphological, physiological and chemical traits

Previous studies have already shown that phenotypic plasticity plays an important role in defense- and growth-related traits of *P*. *lanceolata* [22,47–51]. Changing light conditions for example caused different growth forms in *P*. *lanceolata* as a consequence of phenotypic plasticity [52]. Furthermore, herbivory can function as a driver selecting plant genotypes that express variable resistance traits which in turn comprise resistance to a variety of herbivores [53]. Results from a previous study in the Jena Experiment showed that *P*. *lanceolata* plants suffered higher levels of proportional leaf damage in monocultures than in 60-species mixtures [21] while two other studies in the same experimental grasslands did not find plant diversity effects [19,54]. The weak relationship between plant diversity and herbivory in *P*. *lanceolata* over the course of six years since establishment of the biodiversity experiment might be one possible explanation for similar defense chemistry of plants originating from plant communities with different diversity levels.

Furthermore, six years since the establishment of the experiment may be too short to promote local adaptation. Although the parental generation of plants in our experiment experienced heterogeneous biotic and abiotic environments along the plant diversity gradient, the small spatial scale of our experiment (plot size 20 x 20 m) may have retarded genetic differentiation for a self-incompatible, wind- or insect-pollinated plant such as *P*. *lanceolata*. Under these fine grained environmental conditions the expression of phenotypic plasticity may be more likely than local adaptation *via* genetic differentiation [55]. Our findings are supported by a reciprocal transplant-replant experiment with seed families of *P*. *lanceolata* collected in monocultures and 60-species mixtures of the Jena Experiment which showed that the original plant environment did not affect morphological traits, survival and herbivore leaf damage levels, while the actual growth environment had significant effects on these variables [21].

### Effects of light and nutrient availability on iridoid glycosides and verbascoside concentrations

Plant species richness and plant functional group composition may have a strong impact on light and nutrient availability in a plant community [17,40,56]. Leaf area index and plant biomass increase with increasing plant species richness in experimental grasslands [57,58]. Thus plant diversity can indirectly influence the defense metabolite levels of plants *via* changes in light and nutrient conditions [19].

Under high light conditions in our greenhouse experiment, the concentrations of the iridoid glycosides and verbascoside were highest. This is in line with results from several studies on herbaceous and woody plant species [4,9,59]. Biomass production in the high light and high nutrient environment was increased 8.5 fold compared to plants grown in the low light environment. A reduction in light availability decreased light-saturated rates of photosynthesis of *P*. *lanceolata* irrespective of nutrient availability (Fig 4). The variation in nitrogen concentration in above- and belowground plant tissue was also strongly affected by light availability, while the effect of fertilization was less prominent (Fig 5). The light-dependent usage of CO_2_ for synthesis of sugars, organic acids, and amino acids, and finally the accumulation of biomass is strongly dependent on nitrogen availability in plants [60]. When nutrient levels are increased from moderate to high, photosynthetically fixed carbon is allocated to growth rather than defense or storage compounds and plant growth increases [61].

While we found consistent positive effects of light on *P*. *lanceolata* defense compounds, the effects of nutrient availability on verbascoside and iridoid glycoside concentrations differed: Verbascoside concentrations decreased under high nutrient conditions in our experiment (Fig 2) and also in other studies [1], but iridoid glycoside concentrations were only marginally affected by nutrient availability (Fig 1). Phenolics such as verbascoside are derived from the shikimic acid pathway, whereas terpenoids, including the monoterpene-derived iridoid glycosides, are synthesized via the methylerythritol-phosphate (MEP) pathway [62,63]. Therefore, our results suggest that nutrient levels differentially regulate these two biosynthetic pathways.

Carbon allocation to defense and storage compounds is non-linearly related to nutrient availability [61]. Our data show that nutrient availability can affect secondary metabolism in *P*. *lanceolata*. However, stronger changes in iridoid glycosides and verbascoside as the major defense compounds of this species were visible upon alteration in light availability [1].

### Ecological relevance

We could show that the investment of *P*. *lanceolata* into secondary metabolites varies in response to light and nutrient availability. Our experiment indicates that the availability of nutrients had smaller effects on iridoid glycosides and verbascoside concentrations than light availability. Most likely changes in *P*. *lanceolata* defense chemistry caused by altered abiotic conditions have an effect on invertebrate herbivores. From the literature we know that verbascoside can function against the infestation by microbes or mollusk herbivores [32,64,65], while iridoid glycosides are well known to defend *P*. *lanceolata* against generalist insect herbivores and infestation by bacteria or fungi [23,66,67]. As increased concentration of iridoid glycosides and verbascoside affect herbivores negatively [24,64,68,69], high light conditions and thus higher levels of these defense metabolites might increase the resistance of *P*. *lanceolata* against a broad range of herbivores and pathogens. On the other hand, iridoid glycosides can stimulate oviposition of specialist insects [26–28] and thus high light conditions could enhance the attack by specialist insect herbivores in these plants. In addition to light and nutrients, other influences such as herbivory or pathogen infestation may alter the reaction norm of plants to abiotic factors [70–73]. It is conceivable that the slight mildew infestation affected the secondary metabolites and nutrients in our experimental plants as it is known that powdery mildew acts as a metabolic sink, draining nutrients from the plants [74]. To our knowledge there is no study investigating the effect of powdery mildew on iridoid glycoside and verbascoside production in *P*. *lanceolata*, however as there is evidence from the literature, that powdery mildew infestation is directly correlated to monoterpene emission [75] it is conceivable that this pathogenic fungus also influences the biosynthesis of monoterpene derived iridoid glycosides.

Future studies should test whether plants exhibit differences in their induced resistance against generalist or specialist antagonists under changing environmental regimes, e.g. indirectly brought about by differences in plant diversity. This is specifically relevant as previous biodiversity studies have shown correlations between invertebrate herbivory and plant species richness in experimental and semi-natural grassland communities [21,54,76] but have yet not fully revealed the underlying mechanisms for these patterns.

Simple common garden experiments are valuable to discriminate whether genetic differences or the plant environment contributes most to the phenotypic variation observed. Since we used seed families collected in the same experiment as Mraja et al. [19], we may conclude that plant-diversity related variation in iridoid glycosides observed in the field was due to phenotypic plasticity under different environmental conditions and not attributable to genetic differentiation in response to the different selection regime in plant communities of varying diversity. Due to its phenotypic plasticity, *P*. *lanceolata* might be able to adjust to changing environmental conditions much faster and more efficiently than *via* adaptation due to genetic differentiation.

## Supporting Information

S1 File **Table A.** Number of sown species and species composition of origin plots of *Plantago lanceolata* seed families in the Jena Experiment. **Table B.** Chemical composition of fertilizer for the low-nutrient and the high-nutrient treatment.
(DOCX)
