# Supplementary material for: Light and Nutrient Dependent Responses in Secondary Metabolites of Plantago lanceolata Offspring Are Due to Phenotypic Plasticity in Experimental Grasslands
Source: PLoS One. 2015 Sep 3;10(9):e0136073. doi: 10.1371/journal.pone.0136073 (PMC4559451; doi:10.1371/journal.pone.0136073)
Supplement: S1 File — Table A. Number of sown species and species composition of origin plots of Plantago lanceolata seed families in the Jena Experiment. Table B. Chemical composition of fertilizer for the low-nutrient and the high-nutrient treatment. (DOCX) [file pone.0136073.s001.docx]

**Supporting Information**

**S1 File**

**Table A: Summary of mixed-effects model analyses of iridoid glycoside and verbascoside concentrations in leaves and roots of *Plantago lanceolata***. Plants were grown at two different levels of nutrient and light availability and originating from seed families collected in experimental communities of different plant diversity. Root samples were analysed for offspring originating from three seed families taken from the monoculture and a 60-species mixture.

| Source of variation | Total Iridoidglycosides | Aucubin | Catalpol | Verbascosides |
| --- | --- | --- | --- | --- |
| **Leaves** |  |  |  |  |
| AIC | 1284.1 | 1182.2 | 1279.4 | 1141.4 |
| **Fixed effects** |  |  |  |  |
| Intercept | 2.123 | 1.491 | 1.557 | 2.255 |
| *Origin environment* |  |  | -- | -- |
| Species richness | -- | -- | -- | -- |
| Legume | -- | -- | -- | -- |
| *Growth environment* |  |  |  |  |
| Nutrients | -- | *-0.100* | 0.154 | -0.394 |
| Light | 1.068 | 1.169 | 0.629 | 1.519 |
| Nutrients x Light | -- | 0.225 | -- | -- |
| **Random effects** |  |  |  |  |
| Seed family (SF) | 0.211 | 0.177 | 0.272 | 0.143 |
| SF x Nutrients | -- | -- | -- | -- |
| SF x Light | -- | -- | -- | -- |
| SF x Nutrients x Light | -- | -- | -- | -- |
| Residual | 0.698 | 0.640 | 0.685 | 0.622 |
| **Roots** |  |  |  |  |
| AIC | 732.5 | 33.3 | 184.8 | 997.4 |
| **Fixed effects** |  |  |  |  |
| Intercept | 3.997 | 2.046 | 0.524 | 48.086 |
| *Growth environment* |  |  |  |  |
| Nutrients | -- | -- | *-0.445* | -- |
| Light | 23.735 | 1.166 | 1.264 | -- |
| Nutrients x Light | -- | -- | 0.661 | -- |
| **Random effects** |  |  |  |  |
| Seed family (SF) | <0.001 | <0.001 | 0.290 | 8.330 |
| SF x Nutrients | -- | -- | 0.254 | -- |
| SF x Light | -- | -- | -- | 8.912 |
| SF x Nutrients x Light | -- | -- | -- | -- |
| Residual | 5.078 | 0.269 | 0.468 | 14.647 |

Significance of seed family (SF) and interactions of seed family with the experimental factors (SF × Nutrients, SF × Light, SF × Nutrients × Light) were assessed based on the full fixed effect model. Afterwards, the set of fixed effects containing all significant predictors was determined by stepwise inclusion and model comparison. Estimated coefficients and AIC are given for the resulting best model. Intercept and slopes respectively are shown for the fixed effects and estimated standard deviations are given for the random effects.

**Table B: Summary of mixed-effects model analyses of leaf and root biomass and the shoot: root ratio measured for *Plantago lanceolata*.** Plants grown at two different levels of nutrient and light availability and originating from seed families collected in experimental communities of different plant diversity.

| Source of variation | Leaf biomass | Root biomass | Shoot:root ratio |
| --- | --- | --- | --- |
| AIC | -128.8 | 440.9 | 255.7 |
| **Fixed effects** |  |  |  |
| Intercept | 6.053 | 4.112 | 1.936 |
| *Origin environment* |  |  |  |
| Species richness | -- | -- | -- |
| Legume | -0.012 | -- | -- |
| *Growth environment* |  |  |  |
| Nutrients | 0.012 | -0.428 | 0.439 |
| Light | 0.623 | 2.617 | -1.993 |
| Nutrients x Light | 1.249 | 1.104 | 0.147 |
| **Random effects** |  |  |  |
| Seed family (SF) | <0.001 | 0.158 | 0.121 |
| SF x Nutrients | <0.001 | -- | -- |
| SF x Light | 0.196 | 0.141 | 0.125 |
| SF x Nutrients x Light | 0.189 | -- | -- |
| Residual | 0.199 | 0.330 | 0.278 |

Significance of seed family (SF) and interactions of seed family with the experimental factors (SF × Nutrients, SF × Light, SF × Nutrients × Light) were assessed based on the full fixed effect model. Afterwards, the set of fixed effects containing all significant predictors was determined by stepwise inclusion and model comparison. Estimated coefficients and AIC are given for the resulting best model. Intercept and slopes respectively are shown for the fixed effects and estimated standard deviations are given for the random effects.

**Table 3: Summary of mixed-effects model analyses of maximum rates of photosynthesis (A_max_) and nitrogen concentrations in leaves and roots of *Plantago lanceolata***. Plants were grown at two different levels of nutrient and light availability and originating from three seed families taken from the monoculture and a 60-species mixture.

| Source of variation | A_max_ | Leaf nitrogen | Root nitrogen |
| --- | --- | --- | --- |
| AIC | 273.3 | 371.2 | -7.6 |
| **Fixed effects** |  |  |  |
| Intercept | 6.309 | 38.177 | 2.872 |
| *Growth environment* |  |  |  |
| Nutrients | -0.430 | 17.176 | 0.415 |
| Light | 3.303 | -25.711 | -1.161 |
| Nutrients x Light | 2.056 | -11.370 | -0.271 |
| **Random effects** |  |  |  |
| Seed family (SF) | 0.018 | 3.402 | 0.037 |
| SF x Nutrients | -- | 2.677 | -- |
| SF x Light | 0.932 | 4.301 | -- |
| SF x Nutrients x Light | -- | 4.496 | -- |
| Residual | 1.365 | 2.215 | 0.208 |

Significance of seed family (SF) and interactions of seed family with the experimental factors (SF × Nutrients, SF × Light, SF × Nutrients × Light) were assessed based on the full fixed effect model. Afterwards, the set of fixed effects containing all significant predictors was determined by stepwise inclusion and model comparison. Estimated coefficients and AIC are given for the resulting best model. Intercept and slopes respectively are shown for the fixed effects and estimated standard deviations are given for the random effects.
